# The Study of Using 3D Scan Technique to Evaluate the Expanding Method of Ear Reconstruction Before Operation

**DOI:** 10.1007/s00266-019-01453-y

**Published:** 2019-07-22

**Authors:** Rui Wan, Weiguo Xie, Zhongwei Li, Jingrui Zhou

**Affiliations:** 1 Plastic Surgery Department of Zhongnan Hospital of Wuhan University, Wuhan, Hubei Province China; 2 Plastic Surgery Department of Wuhan Third Hospital, Wuhan, Hubei Province China; 3 State Key Laboratory of Material Processing and Die & Mould Technology in Huazhong University of Science and Technology, Wuhan, Hubei Province China

**Keywords:** 3D scan, Expanding, Microtia, Ear reconstruction

## Abstract

**Purpose:**

Three-dimensional scanning technology was used to measure the expansion of the area and size of auricular skin to meet the normal standard of the external ear before ear reconstruction among microtia patients.

**Materials and Methods:**

The skin surface area of microtia patients was measured by three-dimensional scanner: the surface area (*S*), vertical length (*A*), vertical curve length (*B*), transverse length (*C*), transverse curve length data (*D*), and then taking the average. Corresponding measurements in healthy adults were also obtained: surface area (*S*_0_), the vertical curve length (*B*_0_), and transverse curve length (*D*_0_) of the normal external ear were obtained by scanning normal adult male ears with reference to the range of the vertical length and the transverse straight length. Mean surface area (*S* and *S*_0_), vertical curve length (*B* and *B*_0_), and transverse curve length (*D* and *D*_0_) were compared between microtia patients and healthy adults.

**Results:**

The surface area, vertical curve length, and transverse curve length were statistically significantly higher among healthy adults.

**Conclusions:**

With the amount of expanded water injection of 120–130 ml, the expanded skin still does not reach the standard of the normal external ear in terms of skin surface area and size.

**Level of Evidence IV:**

This journal requires that authors assign a level of evidence to each article. For a full description of these Evidence-Based Medicine ratings, please refer to the Table of Contents or the online Instructions to Authors www.springer.com/00266.

## Introduction

Microtia ear reconstruction is one of the most challenging operations in plastic surgery. Its core is the ear framework and the covering skin. The current mainstream method uses autologous rib cartilage to complete the ear reconstruction. The application of the expander is one of the main surgical procedures. Some scholars use the expander + skin grafting technique. Some other scholars only use the expander and no skin grafting technique. The author uses three-dimensional scanning technology to measure the expanded skin area and evaluate whether the expanded area meets the normal ear’s standards, so as to determine whether a skin graft is needed to supply the area and size.

## Patients and Methods

### Clinical Information

*Patient data* From June 2015 to October 2017, 22 male patients with microtia who were admitted to our hospital were recruited in this study. The participants were 8–24 years old, with an average of 15.3 years. A 50-ml kidney-shaped expander was embedded in the posterior mastoid region, and water injection was 120–130 ml with an average of 123.7 ml.

*Normal external ear data* 30 healthy male adults were randomly selected during the same time period, aged 22–30 years, with an average age of 25.6 years. The criteria for normal ear were defined as: (1) no protruding ear, (2) no cup ear, (3) no cryptotia, (4) no Stahl’s ear or other external ear malformations. The participants were asked to wear short hair to reduce measurement error in the area. Only one side of the external ear was measured.

*3D scanner* The ReadyScan^TM^Pro 3D scanner is independently developed with a high-precision camera calibration algorithm with a calibration accuracy of 0.0367 mm [1]. The data were analyzed with the computer software at this national key laboratory.

### Methods

#### Scanning and Statistical Processing

**Fig. 1:**
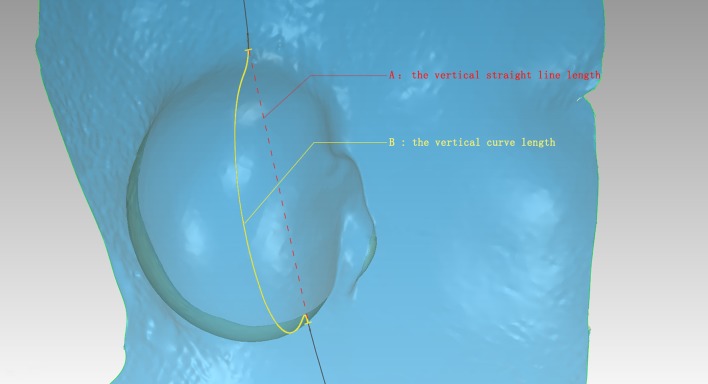
Dimensional *A* and *B* lines after 3D scanning

**Fig. 2:**
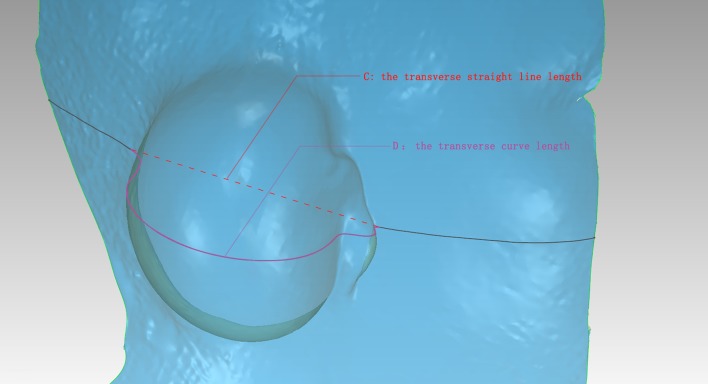
Dimensional *C* and *D* lines after 3D scanning

**Fig. 3:**
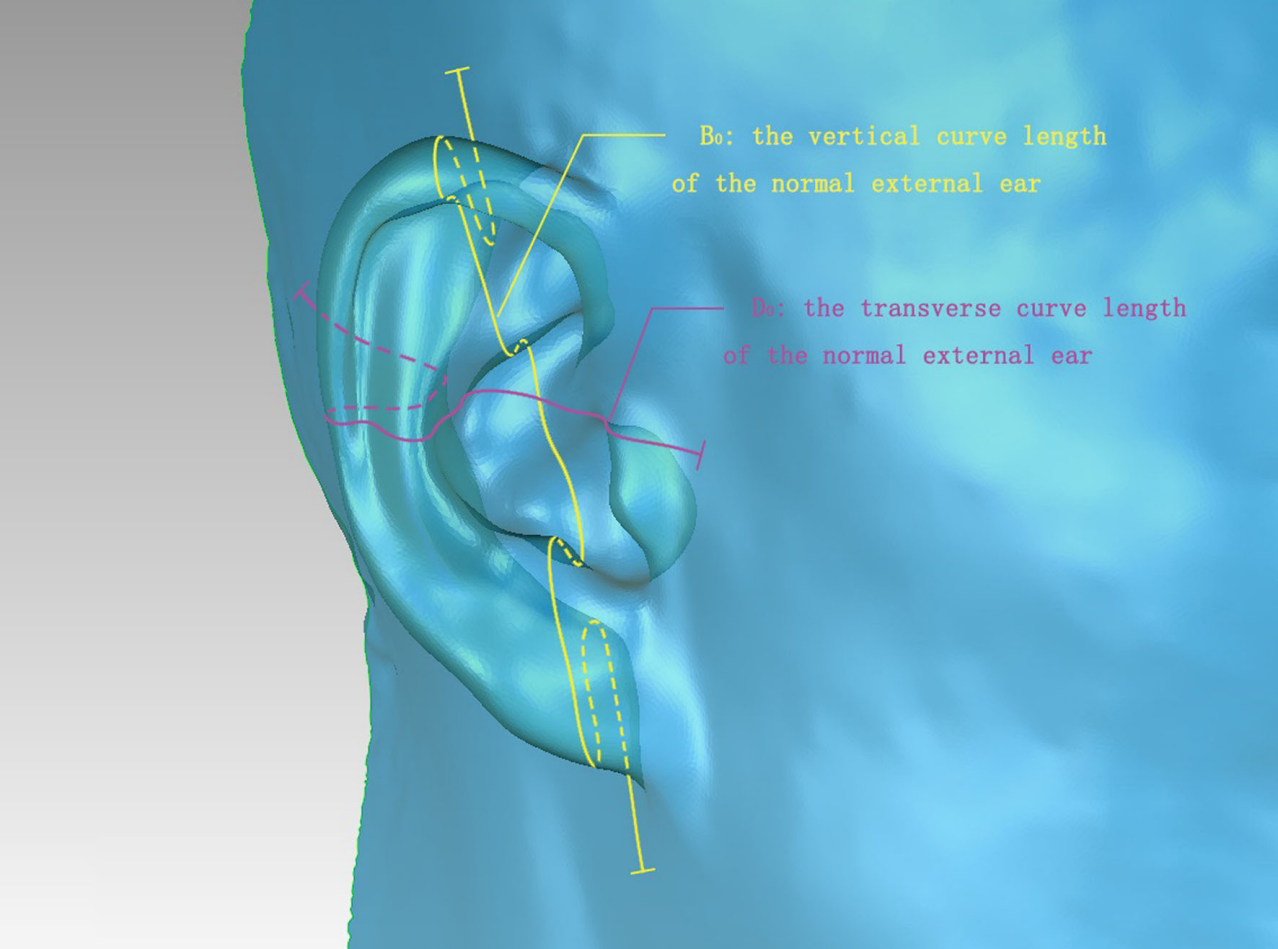
Dimensional *B*_0_ and *D*_0_ lines after 3D scanning the normal external ear

After scanning the microtia patients with implanted expanders, the expanded surface area (*S*), the vertical straight line length (*A*), the vertical curve length (*B*), the transverse straight line length (*C*), and the transverse curve length (*D*) were obtained. For the normal adults, the scanning was performed based on the average value of the vertical straight line length and the transverse straight line as the reference coordinates and the reference range as shown in Figs. 1, 2, and 3.

The data for both patients and healthy adults were entered into the Prism 6.0 statistical software, and *S* and *S*_0_, *B* and *B*_0_, and *D* and *D*_0_ were subjected to independent* t* test comparison.

## Results

The scan data are shown in Tables 1 and 2. The statistical comparison results are shown in Table 3.

**Table 1:** Expanded area scanning data (*S* unit mm^2^; *A*, *B*, *C*, *D* unit mm)

Case	*S*	*A*	*B*	*C*	*D*
1	8439.8	90.0	132.6	72.3	105.7
2	7372.7	77.7	110.4	60.0	101.1
3	10,470.2	88.4	133.7	73.9	133.5
4	8769.0	88.5	126.1	73.7	114.5
5	8693.4	88.0	124.3	73.1	112.8
6	8758.7	88.1	125.1	73.3	113.2
7	8620.9	86.2	123.8	72.3	112.9
8	8790.1	88.9	125.9	74.1	115.0
9	8761.0	88.3	125.6	73.4	113.5
10	8755.8	88.2	125.4	73.2	112.9
11	8762.1	88.3	125.7	73.5	113.5
12	8765.3	88.5	125.8	73.5	113.9
13	8602.7	87.9	123.6	72.0	111.5
14	8903.6	88.5	126.7	73.9	115.1
15	9173.2	89.8	127.5	74.0	116.1
16	8527.6	87.9	123.7	72.9	110.8
17	8763.4	88.3	125.9	73.7	112.9
18	9021.7	88.7	125.8	73.5	114.0
19	8672.5	88.4	124.7	73.1	112.8
20	8730.8	88.3	125.9	73.9	113.5
21	8698.2	88.1	124.8	73.1	112.8
22	8997.4	88.9	126.2	74.3	115.2
Average value	8775.0	88.4	125.4	72.8	113.5

**Table 2:** Normal external ear scanning data (*S*_0_ unit mm^2^; *B*_0_, *D*_0_ unit mm)

Case	*S*_0_	*B*_0_	*D*_0_
1	9540.3	196.7	125.5
2	9225.7	159.1	122.5
3	9097.5	181.5	121.5
4	8824.1	187.7	129.9
5	9218.3	182.1	125.2
6	9180.1	182.3	125.6
7	9081.7	180.9	123.7
8	9337.2	183.5	126.9
9	9281.6	182.7	124.9
10	9170.5	182.0	125.1
11	9390.7	184.1	126.2
12	9270.3	183.9	125.7
13	9217.5	182.0	125.5
14	9324.7	182.9	125.2
15	9321.1	181.8	124.9
16	9217.4	181.5	125.0
17	9160.9	180.4	123.9
18	9173.7	181.2	124.5
19	9337.6	183.4	125.3
20	9478.1	185.9	126.1
21	9501.7	184.7	126.3
22	9203.4	183.0	125.7
23	9510.7	190.2	126.1
24	9153.2	181.2	124.7
25	9079.4	181.3	124.8
26	9183.5	181.3	124.9
27	9257.1	182.4	125.7
28	9451.6	183.1	124.2
29	9309.3	182.5	125.3
30	9321.7	183.0	125.9
Average value	9260.7	182.6	125.2

**Table 3:** Statistical comparison results

Statistics	*S*_0_ versus *S*	*B*_0_ versus *B*	*D*_0_ versus *D*
*P* value	< 0.0001	< 0.0001	< 0.0001

## Discussion

The two most important factors for ear reconstruction are the ear framework and soft tissue coverage. Patients with microtia have limited skin behind the ear. Different scholars use different methods for external ear reconstruction. Brent [2], Nagata [3–5], and Firmin [6] suggest not to expand. Park [7] uses the expander and skin graft methods. In China, Zhuang [8] also uses the expander and skin graft methods. Guo [9] uses the method of expansion without skin graft, and the total water injection amount is 110–120 ml, which achieved a certain good ear reconstruction effect. However, does the expanded skin area behind the remnant meet the size requirements for covering the ear framework? Does the expansion of the skin meet the size requirements for covering the ear framework in the vertical length and transverse length? There are always academic different opinions. The author uses three-dimensional scanning to accurately measure the area and dimension of the expanded skin, to evaluate whether the soft tissue coverage of the ear framework is sufficient and reasonable.

Our results show that the expanded area is significantly smaller than the normal external ear skin coverage area. This means that although the volume of water injection has been quite large, 110–120 ml, the area is still insufficient to totally cover the ear framework transplanted. In addition, after removing the expander, the expanded flap has a certain immediate retraction rate [10]. Under the guidance of plastic surgery: the principle of no tension, the tension of the expanded flap covering the grafted ear framework should not be too large. So from this perspective, the expanded skin area is further insufficient. If you continue to increase the amount of water to expand the skin area, although the area is increased, the vertical straight line length and the transverse straight line length are also increased. And the surface area corresponding to the transverse and vertical length of the normal external ear is increased too; therefore, the expansion of effective area is very limited. Furthermore, the over-expanded skin behind the ear may have some “stretch mark”-like change caused by the breaks of the elastic fiber. Even if the operation is performed and the excessively thin expanded skin covers the ear framework, the reconstructed ear is stiff and weird shape and far from natural looking.

The statistical analysis shows that the transverse curve length and the vertical curve length are also different from the normal external ear. During the reconstruction of the external ear, the transverse protruding height of the reconstructed ear is an important target to achieve. When the reconstructed ear is erected, the shape can appear three-dimensional. The study data show that the average transverse curve length is 11.7 mm less than that of the normal external ear. After the expander is removed, the expanded flap immediately retracts, and the transverse curve length gap becomes larger. If the exogenous skin is not replenished, then the transverse protruding height of the reconstructed ear has to be sacrificed. Some scholars use transverse *V*–*Y* flaps to increase the transverse curve length during surgery, which supplies the transverse curve length by removing the vertical direction skin. However, our study shows that the expanded vertical curve length is also significantly shorter than the vertical curve length of the normal external ear. Therefore, using a *V*–*Y* flap to supply the deficiency of the transverse curve length will actually increase the deficiency in the vertical direction.

We chose the vertical curve length by referring to the physiognomic ear length, that is, the straight line from the top of the ear to the lowest point of the ear lobe, which is generally parallel to the dorsum of the nose. On this straight line, the computer automatically projects onto the ear sulcus surface to obtain the vertical curve length. In the vertical direction, the average value of the expanded skin vertical curve length is 57.2 mm. It is shorter than that of the normal external ear. And the length difference is large. During the operation, the residual ear is removed to form a new earlobe, and the vertical curve length will increase. The preoperative three-dimensional scanning cannot accurately locate the incision, so that the vertical curve length cannot be accurately predicted before the new earlobe is reconstructed. But it is also impossible for the vertical curve length of the reconstructed earlobe to reach 6 cm, because only judged by the naked eye, it is impossible for the vertical length of the normal external earlobe to reach 3 cm.

Because the male and the female appearances are different in the ear length [11], the author selected males with normal external ears to avoid systematic errors caused by gender differences. The author selected normal adult male external ears as the control group. The healthy ears of the microtia patients were not used as the control group. Firstly, the reason is that the healthy ear of microtia patients sometimes had small deformities, such as flaring ear and cup ear. Secondly, the ear in childhood will grow to a certain extent with age [12], and the family members of the patient also hope that the reconstructed external ear could be the same size or close to adulthood.

The mastoid skin thickness of Asian microtia patients is suitable for large tissue skin expansion. Firstly, Asian skin is thicker than Caucasian. As we know, tissue skin expansion for microtia reconstruction began to boom in the 1990s, and the boom disappeared quickly. But from that time to now, this method has become very common in China. In my experience, the expander extrusion rate is very low. Wan [13] reported that there were 3 expander extrusion cases in 308 microtia cases between 2004 and 2016. Secondly, when separating the thin mastoid subcutaneous skin, the separating layer is between the subcutaneous fat and the posterior auricular fascia. If necessary, the flap should be separated under the posterior auricular fascia to ensure sufficient thickness for expansion.

In general, the size of the expanded skin behind the ear with 120–130 ml water injection is still smaller than that of the normal external ear in terms of area, transverse and vertical curve length. The amount of 120–130 ml of water injection is not enough for external ear reconstruction. The expansion of water injection needs to be further increased, and it is possible to expand the skin area to reach the area and size of the normal external ear. The author will write an additional article to report his research.
